# Draft Genome Sequence of Plant Growth-Promoting Endophytic *Microbacterium hydrothermale* BPSAC84, Isolated from the Medicinal Plant *Mirabilis jalapa*

**DOI:** 10.1128/MRA.00406-19

**Published:** 2019-05-30

**Authors:** Ajit Kumar Passari, Vinay Rajput, Zothanpuia, Lakshmi P. M. Priya, Mahesh Dharne, Syed Dastager, Oommen K. Mathew, Abeer Hashem, Elsayed Fathi Abd_ Allah, Bhim Pratap Singh

**Affiliations:** a Department of Biotechnology, Aizawl, Mizoram University, Mizoram, India; b NCIM Resource Centre, CSIR–National Chemical Laboratory, Pune, India; c AgriGenome Labs Pvt. Ltd., Kochi, India; d Botany and Microbiology Department, College of Science, King Saud University, Riyadh, Saudi Arabia; e Mycology and Plant Disease Survey Department, Plant Pathology Research Institute, Agriculture Research Center, Giza, Egypt; f Plant Production Department, College of Food and Agriculture Science, King Saud University, Riyadh, Saudi Arabia; University of Maryland School of Medicine

## Abstract

Endophytic Microbacterium hydrothermale strain BPSAC84, which has antimicrobial potential, was isolated from root tissues of Mirabilis jalapa in Mizoram, Northeast India. The draft genome consists of 3.58 Mb and 3,444 protein-coding sequences.

## ANNOUNCEMENT

Endophytic microbes reside within the plant tissues and have either a symbiotic or mutualistic relationship with their host plant, without causing disease (1). *Microbacterium hydrothermale* strain BPSAC84 was obtained from root tissues of *Mirabilis jalapa* cultivated in Mizoram, Northeast India. The root tissues were surface sterilized, and the fragments were placed on tap water yeast extract agar (TWYE) medium supplemented with nystatin and cycloheximide (60 μg/ml) to suppress fungal growth. The TWYE plate was incubated at 28°C for 2 to 4 weeks (2). Strain BPSAC84 showed moderate growth on the medium, and the colony was observed to have a light-orange color. Based on 16S rRNA sequence analysis, strain BPSAC84 closely matches *Microbacterium hydrothermale* strain 0704C9-2 (GenBank accession number MK696251), with 99.7% similarity. Isolate BPSAC84 showed antimicrobial activity against Staphylococcus aureus, Pseudomonas aeruginosa, Escherichia coli, and Candida albicans. In addition, *Microbacterium hydrothermale* strain BPSAC84 exhibited antibiotic susceptibility to gentamicin, tetracycline, and erythromycin (2). Here, we present the draft genome sequence of *Microbacterium hydrothermale* strain BPSAC84.

A single colony was transferred to 10 ml of tryptone broth (ISP1 broth) and incubated at 28°C for 7 days at 150 rpm. Genomic DNA was extracted using a PureLink genomic DNA isolation kit (catalog number K182002; Invitrogen, Thermo Scientific). Good-quality genomic DNA (500 ng) was fragmented using an M220 instrument (Covaris, Inc.). Fragmented genomic DNA was end repaired and further processed for ligation of Illumina adaptors with an NEBNext Ultra DNA library preparation kit (catalog number E7370L) per the recommendations of the manufacturer. The adaptor-ligated enriched library was purified using AMPure XP beads. Library size distribution was checked on an Agilent TapeStation D1000 DNA chip (product number 5067-5583). The Microbacterium hydrothermale strain BPSAC84 genomic DNA was sequenced with the 2 × 150-bp paired-end read length sequencing protocol of the Illumina MiSeq platform. The quality check of the reads was done using FastQC (3), and the generated sequencing reads were filtered to remove low-quality reads using Trim Galore v0.5.0 (4) with set default parameters. Unicycler v0.4.8 (5) was used for *de novo* assembly with an *N*_50_ value of 503,505 bp. The *Microbacterium hydrothermale* BPSAC84 draft genome sequence contains 18 contigs with an average GC content of 70.97% and a total estimated size of 3,580,540 bp.

The *Microbacterium hydrothermale* strain BPSAC84 genome was annotated on the PATRIC Web server (6) using the Rapid Annotation using Subsystems Technology tool kit (RASTtk) (7), which contains 3,444 protein-coding sequences (CDS), 46 tRNA genes, and 3 rRNA genes. This genome was annotated using genetic code 11, and the taxonomy of this genome is as follows: cellular organisms > *Bacteria* > *Terrabacteria* group > *Actinobacteria* > *Micrococcales* > *Microbacteriaceae* > *Microbacterium* > Microbacterium hydrothermale. The annotation of this strain consists of 1,054 hypothetical proteins and 2,390 proteins with functional assignments. The proteins with functional assignments included 857 proteins with Enzyme Commission (EC) numbers, 742 with gene ontology (GO) assignments, and 655 proteins that were mapped to Kyoto Encyclopedia of Genes and Genomes (KEGG) pathways.

### Data availability.

This whole-genome shotgun project has been deposited at DDBJ/EMBL/GenBank under the accession number STGM00000000. Raw sequences were deposited in the NCBI SRA database under accession number SRS4500629. The BioSample and BioProject numbers are SAMN11159226 and PRJNA527766, respectively.
